# Epidemiological characteristics of common respiratory infectious diseases in children before and during the COVID-19 epidemic

**DOI:** 10.3389/fped.2023.1212658

**Published:** 2023-08-03

**Authors:** Bing Wang, Xiangzhen Gai, Yuling Han, Yanqin Liu, Yun Zhang, Jing Sun, Miao Liu, Huafeng Yu, Zhenju Peng, Xiaoling Wei, Yuna Chang, Xiang Ma, Zhongtao Gai

**Affiliations:** ^1^ Department of Respiratory Medicine, Children’s Hospital Affiliated to Shandong, University (Jinan Children’s Hospital), Jinan, China; ^2^ School of Public Health, Shandong University, Jinan, China; ^3^ Department of Public Health, Children’s Hospital Affiliated to Shandong, University (Jinan Children’s Hospital), Jinan, China; ^4^ Children’s Research Institute, Children’s Hospital Affiliated to Shandong, University (Jinan Children’s Hospital), Jinan, China

**Keywords:** pertussis, measles, scarlet fever, pulmonary tuberculosis, mumps, influenza, COVID-19, children

## Abstract

**Background:**

Since the outbreak of coronavirus disease 2019 (COVID-19), public's awareness of infection prevention and control has increased overall, and various prevention and control measures have been adopted. These measures may also have a certain impact on the occurrence of other infectious diseases. Therefore, we collected information on children with several respiratory infectious diseases in Jinan Children's Hospital in China from 2016 to 2022 and analyzed their changes.

**Method:**

We collected data on age, sex and number of cases of pertussis, measles, scarlet fever, pulmonary tuberculosis, mumps and influenza, which were diagnosed by clinical and laboratory criteria, from 1 January 2016 to 31 December 2022 in Jinan Children's Hospital in Jinan, Shandong Province, China. Data on the number of people affected by these diseases in China from the Chinese Center for Disease Control and Prevention were compared. Then, we processed the data by using WPS Excel 2019 and SPSS.

**Results:**

A total of 12,225 cases were included in this study in Jinan Children's Hospital, which consisted of 3,688 cases of pertussis (2,200 cases before COVID-19 and 1,488 during COVID-19), 680 cases of measles (650 cases before COVID-19 and 30 during COVID-19), 4,688 cases of scarlet fever (4,001 cases before COVID-19 and 687 during COVID-19), 114 cases of tuberculosis (86 cases before COVID-19 and 28 during COVID-19), 449 cases of mumps (340 cases before COVID-19 and 109 during COVID-19) and 2,606 cases of influenza (1,051 cases before COVID-19 and 1,555 during COVID-19). The numbers of children in the hospital with pertussis, measles, scarlet fever, mumps and influenza decreased substantially during COVID-19 in 2020–2022 compared with numbers in 2016–2019, while numbers of patients in China with all six respiratory infectious diseases, including pulmonary tuberculosis, declined during the pandemic. A rebound of pertussis, scarlet fever and influenza was observed in 2021 and 2022.

**Conclusions:**

The study found that viral pathogens such as those causing measles, mumps and influenza all decreased during the pandemic, after which influenza rebounded. Infection diseases caused by bacteria such as scarlet fever and pertussis also decreased during COVID-19, and then a rebound occurred. However, tuberculosis stayed relatively constant.

## 1. Introduction

An ongoing outbreak of a severe respiratory infectious disease called coronavirus disease 2019 (COVID-19) was discovered in Wuhan, Hubei Province, China, at the end of 2019 (1, 2) and spread throughout the country and worldwide. As of 15 January 2023, according to the statistics released by the World Health Organization (WHO), there have been over 662 million confirmed cases and 6.7 million deaths reported worldwide (3). Recent studies have indicated that children are susceptible to severe acute respiratory syndrome coronavirus 2 (SARS-CoV-2), and some even die as a result of the virus (4, 5). To interrupt the transmission of COVID-19, the WHO and various countries around the world have made many efforts, such as COVID-19 vaccines (6), improving surveillance networks, enabling laboratories to identify the virus, implementing mask wearing and frequent and thorough hand washing measures, paying attention to indoor ventilation and encouraging going out less or maintaining a distance of at least 1 meter (3 feet) in public (7). The Chinese government has also enforced strict control measures since the outbreak of COVID-19 recommended by the Center for Disease Control and Prevention in China (China CDC) (8). In light of these protective measures, the current situation of other common respiratory tract infectious diseases, such as pertussis, measles, scarlet fever, pulmonary tuberculosis, mumps and influenza, has changed somewhat. Therefore, how has the epidemiology of these diseases changed in China? This study aimed to explore the epidemiological characteristics of these common respiratory infectious diseases among children in Jinan Children's Hospital (JCH) and patients in China (CH) before and during the COVID-19 epidemic.

## 2. Methods

### 2.1. General information

This study was conducted in the Children's Hospital affiliated with Shandong University (Jinan Children's Hospital) in Jinan, Shandong Province, China, from 1 January 2016 to 31 December 2022. Shandong Province has the largest population in northern China, and it is one of only two provinces in the country with a population of over 100 million (9). Jinan Children's Hospital is the children's medical centre in Shandong Province, serving children from various parts of the province. There are 1,400 established beds and 40 clinical and medical technology departments, with an annual outpatient volume of more than 1.1 million and 39,000 discharged patients (10). It is a large general hospital integrating medical treatment, teaching, scientific research, prevention, rehabilitation and health care.

### 2.2. Data source

Data on the number of diseases, sex and age among the children with common respiratory infectious diseases in Jinan Children's Hospital from 1 January 2016 to 31 December 2022, including pertussis, measles, scarlet fever, pulmonary tuberculosis, mumps and influenza, were obtained from the public health department in the hospital. The department is an internal department of public medical institutions responsible for public health work. It is responsible for collecting information on different infectious diseases and reporting it to the superior health administrative department. Infectious disease data are based on ICD-10 codes. Because of the lack of test reagents, data on influenza in JCH in 2016 are missing.

The number of people affected by common respiratory infectious diseases in China was collected according to CDC data in China. This database collects all data reported on infectious diseases since the Chinese infectious disease network direct reporting system was implemented in 2004, mainly including the number of patients, age group, sex, and number of deaths, among others. The network reporting system covers 100% of the Centers for Disease Control, 98% of medical and health institutions at and above the county level and 88% of township health centres nationwide (11).

### 2.3. Disease diagnosis

Children were diagnosed with pertussis, measles, scarlet fever, pulmonary tuberculosis, mumps or influenza based on recommendations for the diagnosis and treatment of Chinese paediatric infectious diseases. (1) The criteria for defining a pertussis case were as follows: cough lasting for more than 14 days associated with at least one of the other symptoms: paroxysmal cough, inspiratory whooping and posttussive vomiting. A suspected case of pertussis was considered a confirmed case if the child presented a persistent cough and a positive nasopharyngeal swab culture or a positive throat swab PCR or if the antibody against pertussis toxin (anti-PT) IgG was positive (12). (2) The clinical diagnostic criteria for measles were as follows: ① contact history with measles patients or travelling to high incidence areas of measles within 7–21 days; ② Koplik's spots seen in buccal mucosa at the early stage of the disease. A laboratory-confirmed case was considered when one of the following conditions was met: positive specific IgM antibodies in serum or detection of viral RNA in throat swabs or urine (13). (3) A scarlet fever case was characterized by a red rash after 12–48 h and was usually accompanied by a sore throat and flushed cheeks. Group A *streptococcus* (GAS) was cultured using throat swab materials (14). (4) To define a clinical case of paediatric pulmonary tuberculosis, the following three conditions must be met: ① signs and symptoms related to pulmonary tuberculosis, such as a chronic cough or wheezing, long-term fever, weight loss or failure to thrive; ② chest imaging showing the characteristics of active pulmonary tuberculosis; ③ positive tuberculin skin test (TST) results and/or recombinant *Mycobacterium tuberculosis* fusion protein (EC) results and/or interferon-gamma release assay (IGRA) results. The laboratory criteria of tuberculosis were as follows: positive stains of sputum/sputum induction/bronchoalveolar lavage fluid/gastric aspirates, positive culture of *Mycobacterium tuberculosis* (MTB), positive nucleic acid amplification test (NAAT), or positive pathological examination results (15). (5) The clinical diagnosis of mumps can be based on painful swollen salivary glands, particularly the parotid glands in front of the ears on both sides of the face. The laboratory criteria were isolation of virus from a swab inside the cheek, a positive PCR of the swab, or specific IgM mumps antibodies positive in serum (16). (6) The criteria for defining a clinical case of influenza were abrupt onset of fever, chills or sweats, muscle pain, cough and malaise. Influenza can be diagnosed by laboratory testing when meeting one of the following three criteria: positive rapid molecular assays, a positive viral culture or positive reference laboratory polymerase chain reaction testing (17).

### 2.4. Vaccination

Pertussis, measles, pulmonary tuberculosis, mumps and influenza are all vaccine-preventable diseases; scarlet fever is not. It should be noted that based on the national immunization programme in China, the bacillus Calmette-Guerin (BCG) vaccine against tuberculosis is given to infants at birth. Children receive the diphtheria, tetanus, and acellular pertussis (DTaP) vaccine at the ages of 3–5 months, and a booster dose is given to children at an age of 18 months. Infants receive the measles-rubella (MR) vaccine at the age of 8 months and the measles-mumps-rubella (MMR) vaccine at the age of 18–24 months. In addition, children in Beijing city, Tianjin city, Shanghai city and Shandong Province are given a 2-dose mumps-containing vaccine (MuCV) by the age of 6 years as part of Expanded Program on Immunization (EPI) (18). All vaccines mentioned above are mandatory and free. China recommends that children aged more than 6 months receive the influenza vaccine annually, though the vaccine is optional and paid out of pocket (19). Vaccination may be delayed due to requirements of home quarantine.

### 2.5. Data analysis

WPS Excel 2019 (Beijing Jinshan Office Software Co., Ltd., China) was used to organize the data and draw diagrams such as line charts and bar graphs. The *χ*^2^ test and Fisher's exact test were applied to statistically compare the composition ratios of children with different respiratory infectious diseases in JCH before and during COVID-19 using SPSS version 25.0 (SPSS Inc., Chicago, IL, USA). A cut-off of *P* < 0.05 was considered statistically significant.

## 3. Results

### 3.1. General information

Common respiratory infectious diseases among children were pertussis, measles, scarlet fever, pulmonary tuberculosis, mumps and influenza. A total of 12,225 cases from 1 January 2016, to 31 December 2022, were included in this study at Jinan Children's Hospital. The numbers of children with pertussis, measles, scarlet fever, pulmonary tuberculosis, mumps and influenza before and during COVID-19 were as follows: 2,200 cases and 1,488 cases, 650 cases and 30 cases, 4,001 cases and 687 cases, 86 cases and 28 cases, 340 cases and 109 cases, and 1,051 cases and 1,555 cases, respectively. Scarlet fever, pertussis and influenza were the most common respiratory infectious diseases in this children's hospital. However, based on the data of the China CDC, influenza, tuberculosis and mumps were most common in China (Table 1).

**Table 1:** Number of cases of pertussis, measles, scarlet fever, tuberculosis, mumps and influenza in JCH and CH.

Year diseases		2016	2017	2018	2019	2020	2021	2022	Total
Pertussis	JCH	260	449	799	692	93	135	1,260	2,886
	CH	5,584	10,390	22,466	30,737	4,994	9,162	39,781	123,104
Measles	JCH	596	13	21	20	28	2	0	680
	CH	24,820	5,941	4,483	3,573	1,234	916	981	41,948
Scarlet fever	JCH	1,012	1,068	777	1,144	221	350	116	4,628
	CH	59,282	74,370	79,845	83,028	17,206	29,507	21,216	364,454
Tuberclosis	JCH	25	26	12	23	14	4	10	107
	CH	16,72,472	16,62,779	11,10,659	10,34,760	876,546	828,074	712,586	78,97,876
Mumps	JCH	180	71	43	46	49	25	35	421
	CH	175,001	252,740	261,493	303,105	130,901	120,776	105,108	13,49,124
Influenza	JCH	–	131	160	760	482	164	909	2,112
	CH	306,682	456,718	768,289	35,07,306	12,26,804	654,656	24,67,408	93,87,863

JCH, Jinan children's hospital; CH, China.

### 3.2. Age-related rates of common respiratory infectious diseases in children in the hospital before and during COVID-19

The age distribution of pertussis in JCH showed that more children aged >6 years had pertussis in 2020–2022 than in 2016–2019, and the difference was statistically significant (*χ*^2^ = 312.234, *P *< 0.001). More children aged >8 months had measles during COVID-19 than before the pandemic (*χ*2 = 15.395, *P *< 0.001). The onset of scarlet fever was most frequent at the age of 3–10 years old before and during COVID-19. Pulmonary tuberculosis was distributed among children of all ages between 2016 and 2022. There were many more children aged ≥2 years with mumps from 2016 to 2022 than children aged <2 years. Children aged 1–6 years old were more susceptible to influenza before and during COVID-19 (Table 2).

**Table 2:** Age composition ratio of children with pertussis, measles, scarlet fever, mumps and influenza in JCH from 2016 to 2019 vs. from 2020 to 2022.

Disease	Age	2016–2019	2020–2022	*χ* ^2^	*P*
Pertussis	<1 year	54.3% (1,195/2,200)	35.3% (526/1,488)	128.324	<0.001
	1 year–6 years	41.8% (920/2,200)	41.9% (623/1,488)	0.001	0.976
	>6 years	3.9% (85/2,200)	22.8% (339/1,488)	312.234	<0.001
Measles	≤8 months	56.5% (367/650)	26.7% (8/30)	10.292	0.001
	>8 months	43.5% (283/650)	73.3% (24/30)	15.395	<0.001
Scarlet fever	<3 years	4.6% (184/4,001)	6.1% (42/687)	2.932	0.087
	3 years–10 years	94.7% (3,789/4,001)	93.2% (640/687)	2.673	0.102
	>10 years	0.7% (28/4,001)	0.7% (5/687)	0.007	0.809^a^
Mumps	<2 years	6.5% (22/340)	1.8% (2/109)	3.506	0.061
	≥2 years	93.5% (318/340)	98.2% (107/109)	3.506	0.061
Influenza	<1 year	11.8% (124/1,051)	9.7% (150/1,555)	3.087	0.079
	1 year–6 years	74.7% (785/1,051)	77.2% (1,201/1,555)	2.238	0.135
	>6 years	13.5% (142/1,051)	13.1% (204/1,555)	0.084	0.772

^a^ Fisher's exact test.

Thus, the high incidence rates by age of scarlet fever, pulmonary tuberculosis, mumps and influenza were similar before and during COVID-19 (2016–2019 vs. 2020–2022), except that the proportion of children older than 6 years of age with pertussis increased markedly and the proportion of children aged >8 months with measles accounted for a larger proportion from 2020 to 2022.

### 3.3. Sex data of common respiratory infectious diseases among children in JCH before and during the COVID-19 outbreak

There was overall male predominance regarding the respiratory infectious diseases of pertussis, measles, scarlet fever, mumps and influenza from 2016 to 2022. However, the number of tuberculosis cases was too small to come to a conclusion (Table 3).

**Table 3:** Sex ratio of pertussis, measles, scarlet fever, pulmonary tuberculosis (TB), mumps and influenza from 2016 to 2022 in JCH.

Year disease	Pertussis (Males, %)	Measles (Males, %)	Scarlet fever (Males, %)	Tuberculosis (Males, %)	Mumps (Males, %)	Influenza (Males, %)
2016	152 (58.5)	382 (64.1)	640 (63.2)	16 (64.0)	120 (66.7)	–
2017	242 (53.9)	10 (76.9)	674 (63.1)	18 (69.2)	42 (59.2)	80 (61.1)
2018	410 (51.3)	16 (76.2)	478 (61.5)	4 (33.3)	29 (67.4)	81 (50.6)
2019	375 (54.2)	13 (65.0)	696 (60.8)	11 (47.8)	30 (69.8)	429 (56.4)
2020	46 (49.5)	15 (53.6)	125 (56.6)	1 (7.1)	30 (61.2)	291 (60.4)
2021	69 (51.1)	2 (100.0)	209 (59.7)	3 (75.0)	20 (80.0)	107 (65.2)
2022	707 (56.1)	0 (0.0)	82 (70.7)	8 (80.0)	22 (62.9)	538 (59.2)

### 3.4. Number of different respiratory infectious disease cases by month from 2016 to 2022

**Figure 1:**
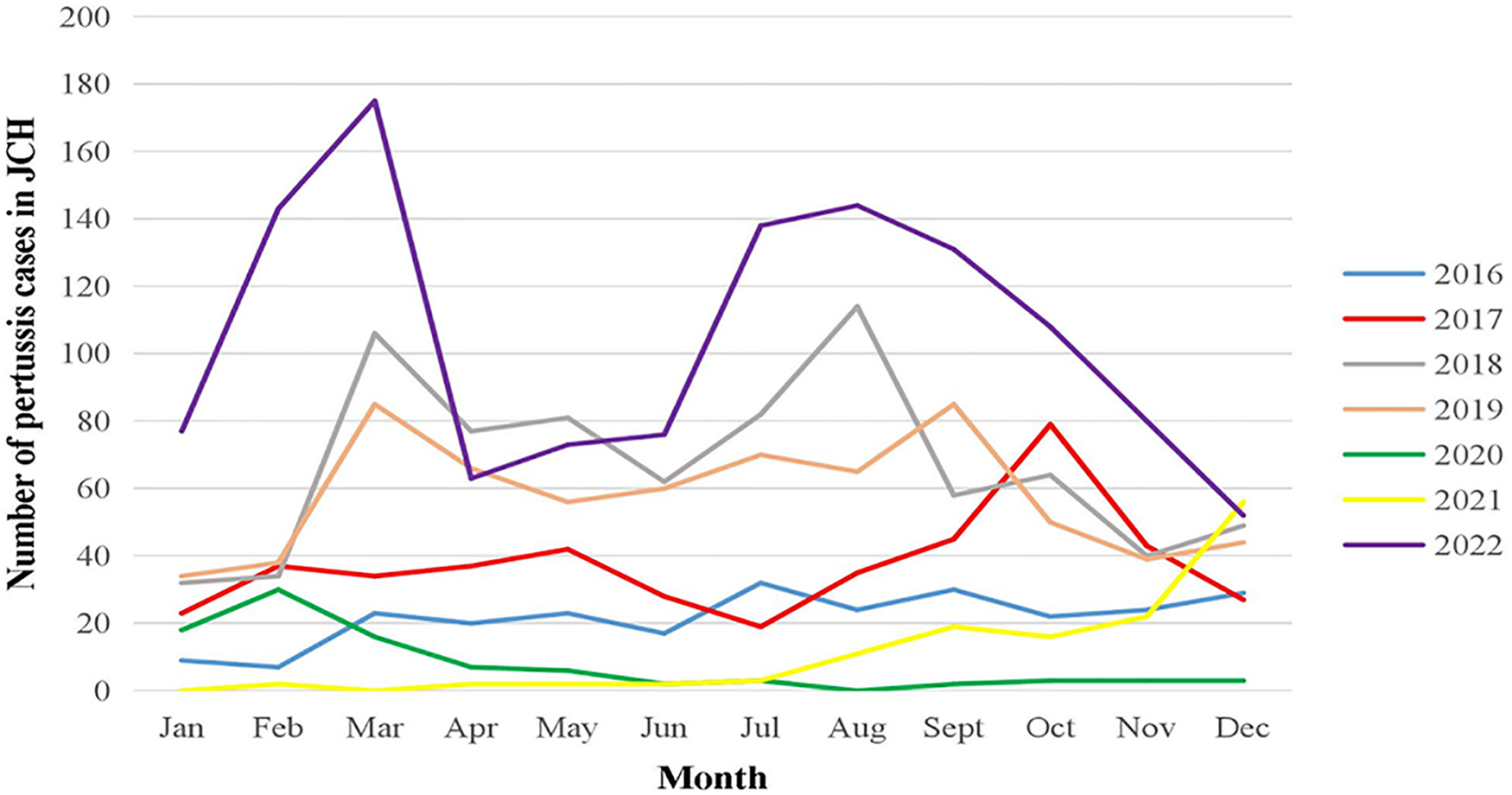
Number of pertussis cases by month in JCH, 2016–2022.

There was an a increase in the number of cases from September to November 2017 in JCH, while CH had a downward trend at the same time according to the data of China CDC. In 2018 and 2019, there was a high incidence of pertussis in both JCH and CH, and there were two peaks of pertussis incidence in JCH in 2018 and 2019 (March to May and September to November), yet there was only one peak in August in CH. During the COVID-19 pandemic (2020 and 2022), the morbidity of pertussis decreased sharply in both JCH and CH compared with that before the epidemic. However, the number of pertussis cases had increased since the beginning of 2022 (Figure 1).

**Figure 2:**
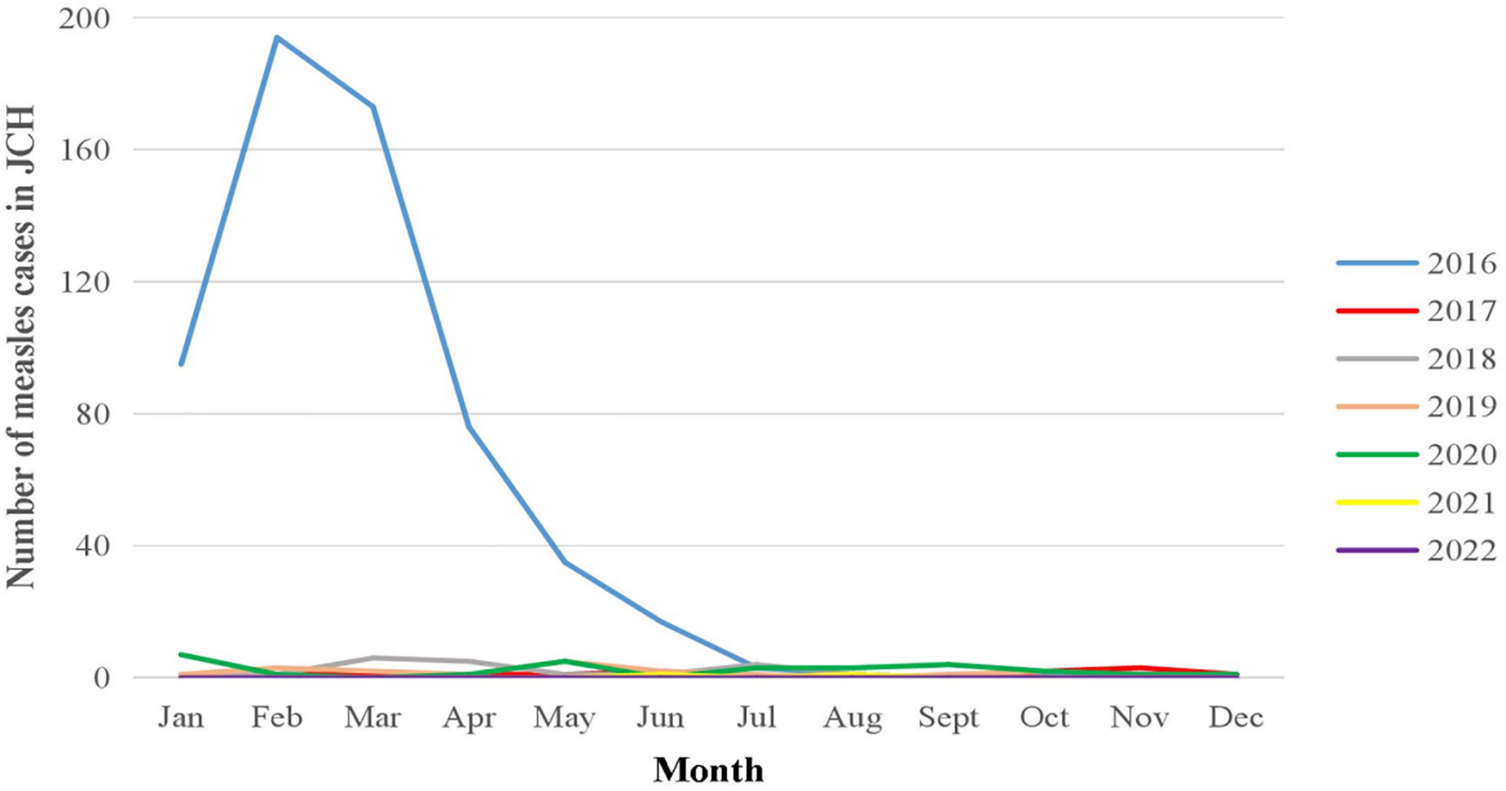
Number of measles cases by month in JCH, 2016–2022.

The first half of 2016, especially spring, was the peak period of measles in JCH and CH. Then, in JCH, the monthly morbidity of measles was similar and remained at a quite low level from 2017 to 2022. However, according to the CH data, there were still outbreaks in the spring from 2017 to 2019, while the trend plateaued during the COVID-19 pandemic from 2020 to 2022 (Figure 2).

**Figure 3:**
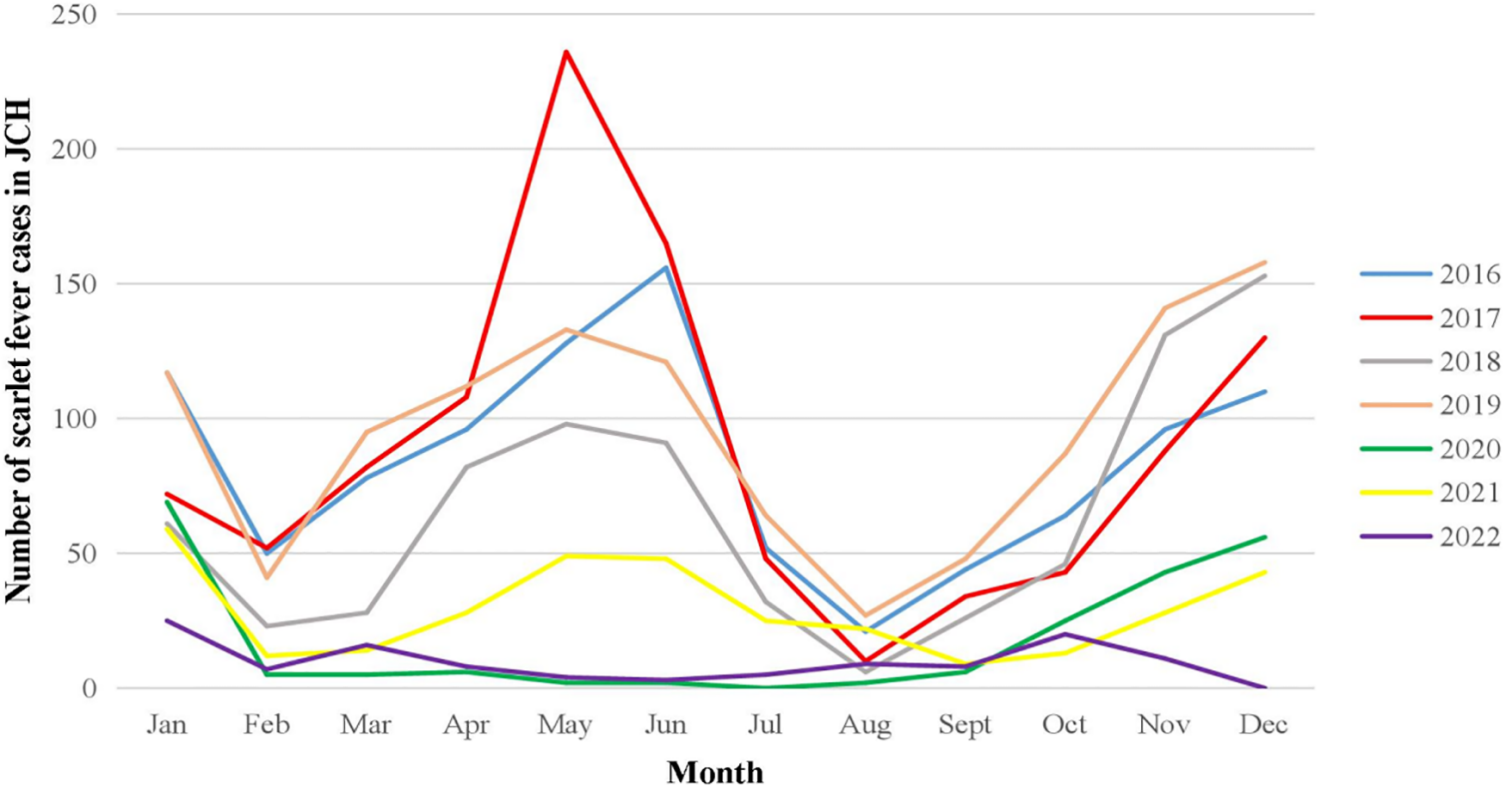
Number of scarlet fever cases by month in JCH, 2016–2022.

The peak seasons of scarlet fever were spring and winter from 2016 to 2019 in both JCH and CH, with scarlet fever occurring mainly from April to June and from November to January of the next year. However, the trend was not as obvious in 2020 when the number of cases was lowest. After 2020, the confirmed number of cases rebounded in 2021 and 2022 (Figure 3).

**Figure 4:**
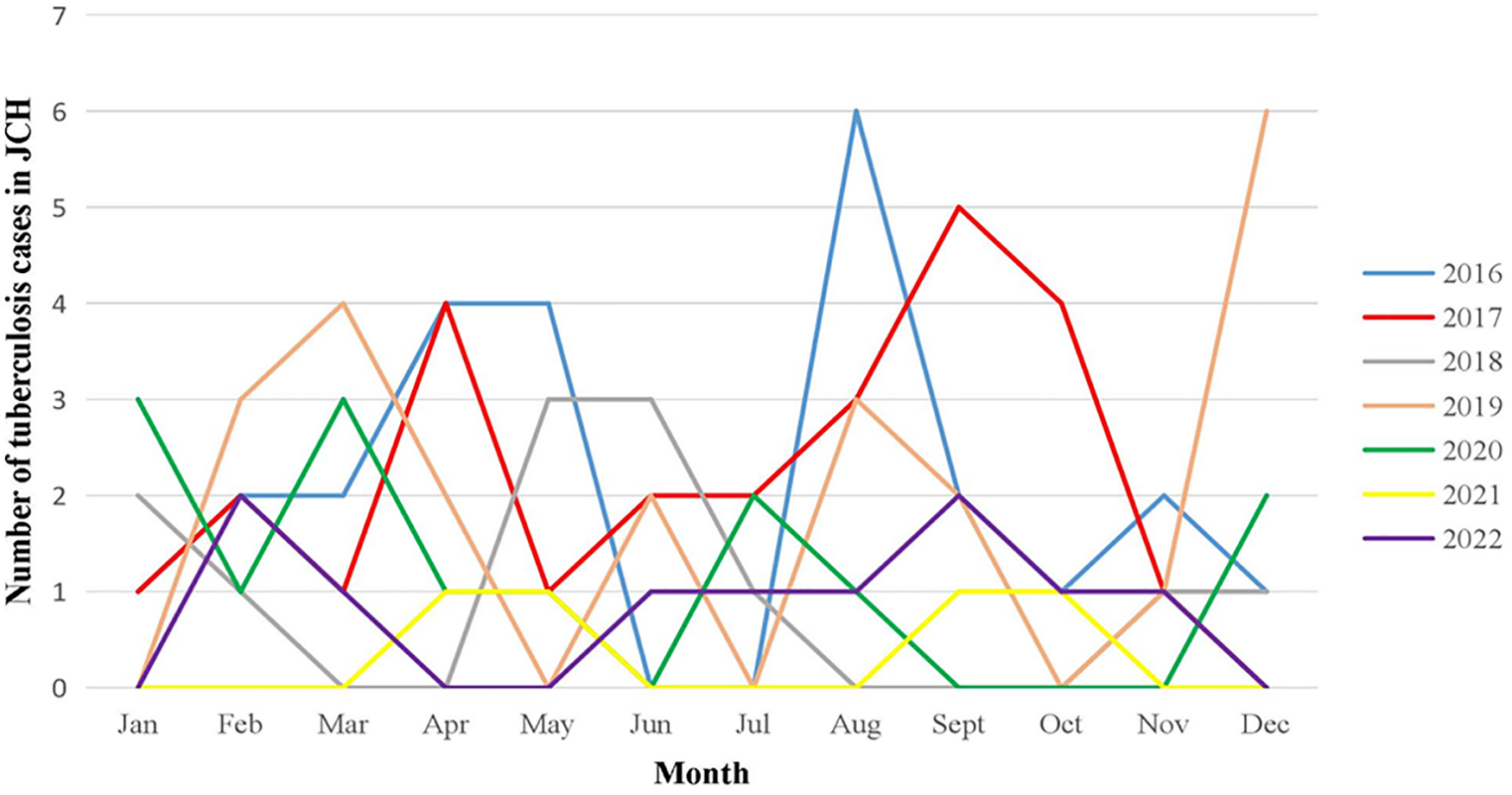
Number of tuberculosis cases by month in JCH, 2016–2022.

The seasonal distribution of tuberculosis was irregular in JCH and CH. The monthly number of children with TB remained in the single digits in JCH from 2016 to 2022. However, in CH, the number of confirmed cases remained quite large every month, ranging from 160,000 to 40,000, and showed a significant downward trend beginning in 2016 according to China CDC data (Figure 4).

**Figure 5:**
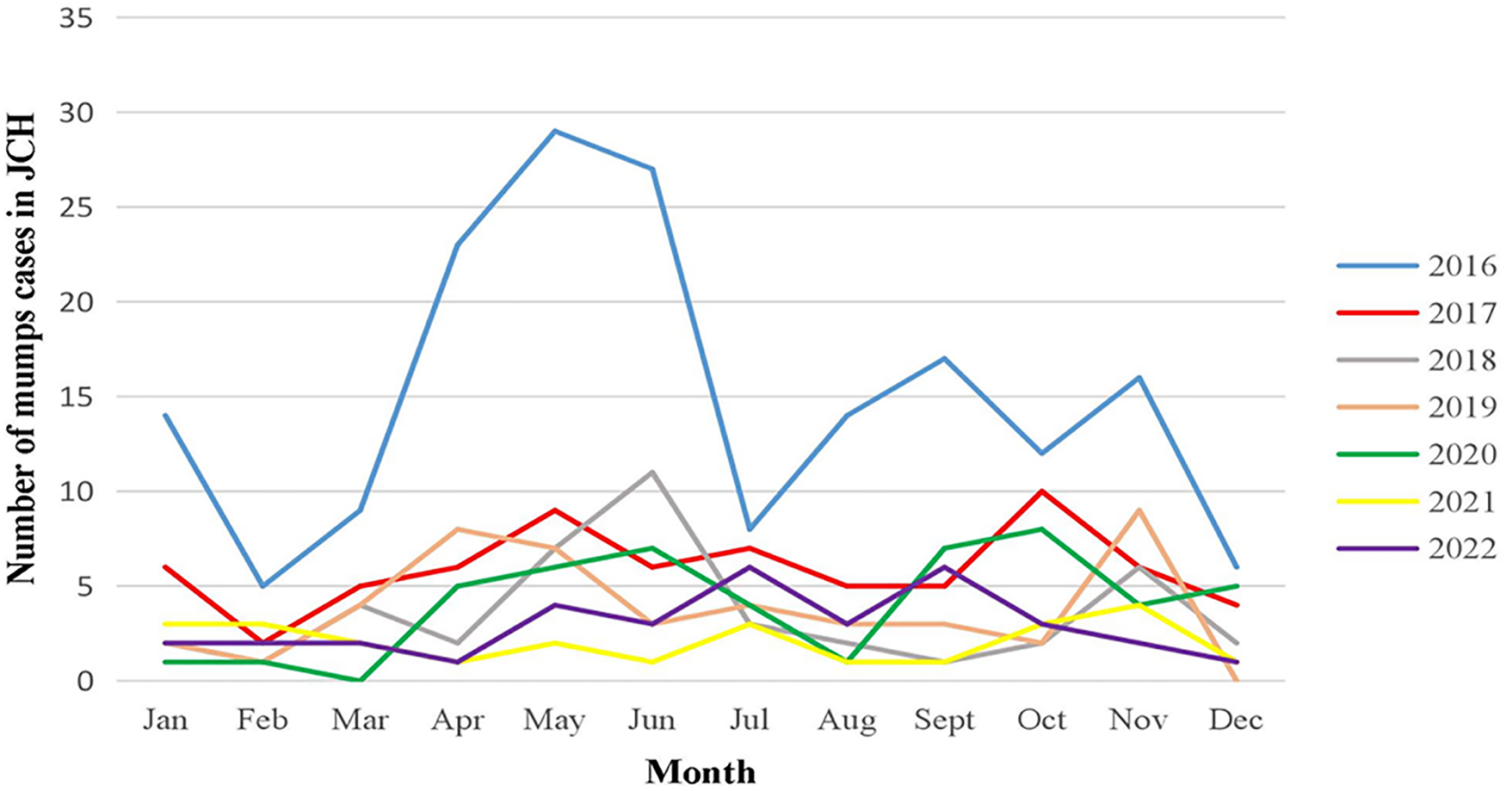
Number of mumps cases by month in JCH, 2016–2022.

April-July and November to the following January were the two peaks of mumps in JCH and CH. Before the epidemic, there were many more children confirmed in 2016 than in the other years in JCH, yet the number of patients in CH increased yearly from 2016 to 2019. The number of mumps cases fell during the COVID-19 epidemic compared with that in 2016–2019 in JCH and CH. However, at the beginning of the epidemic in 2020, the number of mumps cases fell to the lowest point in March in JCH and in April in CH and then increased gradually over the next few months (Figure 5).

**Figure 6:**
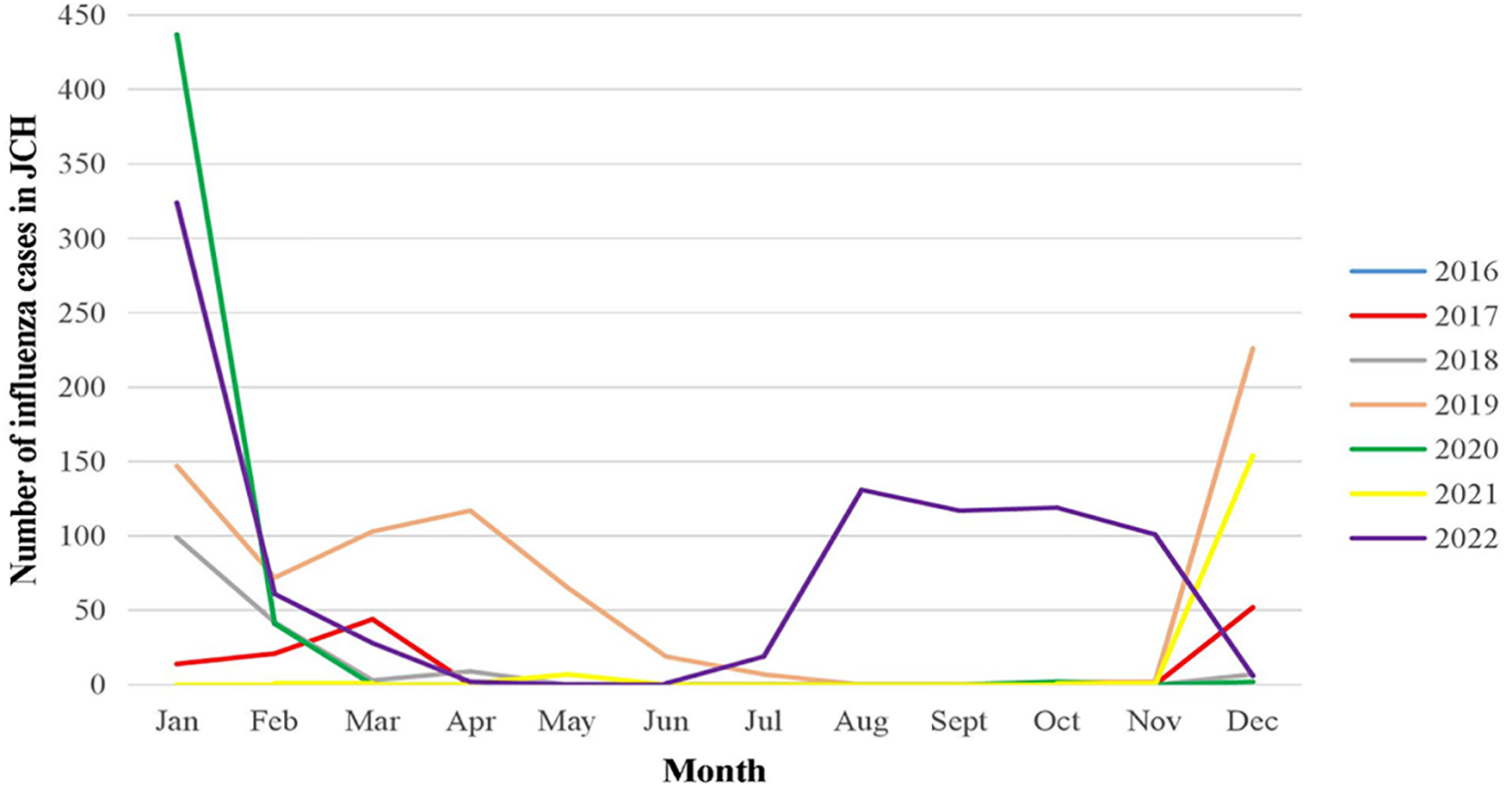
Number of influenza cases by month in JCH, 2017–2022.

Winter and spring were the most common seasons for influenza, and the number of influenza cases increased in January each year in JCH (2017–2019) and CH (2016–2019) during the prepandemic period. However, the number of cases declined sharply at the beginning of 2020 and increased markedly from December 2021 to 2022 (Figure 6).

The numbers of children in JCH with pertussis, measles, scarlet fever, mumps and influenza decreased substantially during COVID-19 from 2020 to 2022 compared with the numbers in 2016–2019, while the numbers of patients with all six respiratory infectious diseases, including pulmonary tuberculosis, declined in CH during the pandemic. In 2022, the peak season of influenza started from the early summer.

## 4. Discussion

Since COVID-19 emerged at the end of 2019, it has caused a large number of infections and deaths, and the number is still increasing daily. In view of the ongoing outbreak and its harmful effects, countries around the world have adopted many measures to interrupt transmission. Thanks to these measures, this study found some changes in the epidemiological characteristics of common respiratory infectious diseases in children.

This study indicated that the numbers of children infected with pertussis, measles, scarlet fever, mumps and influenza in JCH and CH decreased significantly during COVID-19 from 2020 to 2022 compared with the numbers in 2016–2019. This finding was consistent with those of several studies conducted in Taiwan, China, South Korea, Thailand and France (20–23). According to data from the Chinese CDC, the number of patients with all six respiratory infectious diseases, including pulmonary tuberculosis, decreased from 2020 to 2022. This conclusion was consistent with that in Hu's research (24). The main reasons for this phenomenon may be (1) public health measures (mask wearing, hand washing, social distancing measures, school closures, travel restrictions, etc.) taken to fight against COVID-19 that effectively blocked the mode of transmission mainly by reducing human-to-human contact (25). The back-to-school time of primary and secondary schools was delayed to late May and early June in 2020 according to the government notice in Shandong Province (26), and July to August was the summer vacation time, so gatherings were reduced. Scarlet fever and mumps cases gradually increased in September 2020, perhaps because the high-risk ages for the two respiratory infectious diseases were over 3 years and 2 years of age, respectively, when students start attending kindergarten and school. (2) Access to hospitals decreased markedly during the COVID-19 pandemic because people were worried about contracting COVID-19 (27). (3) Medical resources were possibly diverted to COVID-19 testing so that testing opportunities for other infectious diseases were limited.

This study found that the number of pertussis cases increased from 2017 to 2019, highlighting the pertussis resurgence that has occurred in other countries (28). Part of this is due to decreased immunity induced by the pertussis vaccine. In addition, a combination of several other reasons has led to pertussis resurgence, including progress of knowledge about pertussis, confirmation of cases among older children and adults and antigenic variation of strains (29, 30). Therefore, we need to enhance research, protection and control of pertussis to reduce its burden.

Due to the vaccination shedule of measles, more children aged ≤8 months suffered from measles than children aged >8 months between 2016 and 2019. In addition, the number of measles cases in 2016 was more than that in 2017 and 2018 in JCH, which is consistent with the cases reported in the whole country of 1.8 per 100,000 people in 2016, 0.43 per 100,000 people in 2017, and 0.28 per 100,000 people in 2018 (31, 32). However, the measles situation worldwide was still very serious during the COVID-19 pandemic. In 2020, 22.3 million children missed their first dose of measles vaccine (MCV1), which was 3 million more than in 2019, marking the largest increase in twenty years (33). Moreover, critical gaps in disease surveillance, with large numbers of unvaccinated children, have increased the risk of measles outbreaks (34).

Regarding mumps in this study, the two epidemic peaks in JCH and CH were April–July and November to the following January, respectively, and were the same as those in Cui's research conducted in China during 2013–2015 (35). And this study suggested that mumps cases decreased from 2016 to 2019 in JCH located in Shandong Province, while in CH, the number of cases increased so that it peaked in 2019. This was because high incidence age of mumps was over 2 years and only children living in Beijing city, Tianjin city, Shanghai city and Shandong Province could obtain a 2-dose MuCV by the age of 6 years. The rest of China adopted just 1 dose of MuCV. According to the WHO, the coverage rate of MuCV must be above 90% to effectively block the mumps epidemic, so the WHO recommended that 2 doses of the vaccine are required for long-term protection against mumps (36). Therefore, we should promote 2-dose MuCV throughout China to decrease the morbidity of mumps. Moreover, the blocking of the vaccination campaign due to the COVID-19 lockdown could contribute to a greater spread of mumps (37).

Scarlet fever was the most common respiratory infectious disease in children in JCH and no existing vaccines can prevent it. Both this study and Li's research (38) suggested that scarlet fever appeared more often in the spring and autumn seasons. This study indicated that the onset of scarlet fever was most frequent at the age of 3–10 years old. Similarly, a survey in the United Kingdom suggested that 87% of the cases were children aged under 10 years (39). In this study, the number of scarlet fever rebounded in 2021 and 2022. It was consistent with several other studies which highlighted that the rebound was due to the relaxation of public health measures (23, 25).

The cases of tuberculosis among children of different ages were distributed throughout the year during 2016 and 2022 in this study, with fewer than 10 cases every month in JCH. However, the total number of tuberculosis cases in China every month was quite striking, fluctuating by approximately 100,000. Of course, part of this was related to the special management mechanism in China that all suspected TB patients are admitted to specialized tuberculosis hospitals. On the other hand, according to the WHO, children accounted for only 11% of all TB patients in 2020 (37–40), yet the number of child TB cases reported in China accounted for only 1% of cases among people of all ages in our country (41). This meant that tuberculosis was perhaps underrecognized and underreported in children, which occurred in many countries and areas worldwide (42). This is because the diagnosis of pulmonary tuberculosis in children has a certain specificity due to their growth and development and pathogenic characteristics (15). According to the Chinese CDC, the number of TB patients per month has decreased since COVID-19 broke out. Continuing efforts need to be made to formulate prevention and control measures to fight tuberculosis.

This study showed that the upwards trend in influenza was blocked in JCH and CH during the COVID-19 pandemic. According to the WHO, significant declines in influenza virus infection were reported in many countries, such as the United States, Australia and South Korea, during the pandemic (43, 44). However, it should be noted that the number of influenza cases rebounded at the end of 2021, with the peak season starting in early summer in 2022. This result was the same as in Lee's research and prompts new questions regarding the control of influenza: will influenza seasonal patterns return to normal after COVID-19? Should we change the timing of influenza vaccination? What about other respiratory infectious diseases (45)?

On the other hand, because pertussis, measles, pulmonary tuberculosis, mumps and influenza were all belonged to vaccine-preventable diseases, the blocking of the vaccination campaign, as well as the relaxation of comprehensive intervention policies during the pandemic also contributed to the rebound of some diseases, such as pertussis, scarlet fever and influenza in this study. The WHO and United Nations International Children's Emergency Fund (UNICEF) declared that the pandemic led to the largest continued backslide in vaccinations in 30 years, with 25 million infants missing out on lifesaving vaccines in 2021 (46). More efforts need to be made to start the rountine vaccination as soon as possible and to develop a more reasonable immunization program.

Several limitations of this study need to be noted. First, due to the lack of test reagents, the data on influenza in Jinan Children's Hospital in 2016 were missing. Second, this was a single-center study that could not represent the situation of all children in all of China. However, changes in incidence trend of the diseases in our study were similar in JCH and CH. And as the largest children's hospital in Shandong Province with the second largest population (more than 100 million) in China, the annual outpatient number is more than 1.1 million, which is representative to some extent, especially for North China.

## 5. Conclusion

The study collected information on children with several respiratory infectious diseases in Jinan Children's Hospital in China from 2016 to 2022 and analysed their epidemiological changes. We found that viral pathogens such as those causing measles, mumps and influenza all decreased during the pandemic, after which influenza rebounded. Infection diseases caused by bacteria such as scarlet fever and pertussis also decreased during COVID-19, and then a rebound occurred. However, tuberculosis stayed relatively constant. This may also reflect that tuberculosis may preferentially spread within households and that many viral diseases spread in community-and school-based settings.

## Data Availability

The data analyzed in this study is subject to the following licenses/restrictions: The dataset is not available to the manuscript. Requests to access these datasets should be directed to https://www.chinacdc.cn/jkzt/crb/.
